# An approach to identify a minimum and rational proportion of caesarean sections in resource-poor settings: a global network study

**DOI:** 10.1016/S2214-109X(18)30241-9

**Published:** 2018-08

**Authors:** José M Belizán, Nicole Minckas, Elizabeth M McClure, Sarah Saleem, Janet L Moore, Shivaprasad S Goudar, Fabian Esamai, Archana Patel, Elwyn Chomba, Ana L Garces, Fernando Althabe, Margo S Harrison, Nancy F Krebs, Richard J Derman, Waldemar A Carlo, Edward A Liechty, Patricia L Hibberd, Pierre M Buekens, Robert L Goldenberg

**Affiliations:** Institute for Clinical Effectiveness, Buenos Aires, Argentina (J M Belizán PhD, N Minckas MSc, F Althabe MD); RTI International, Durham, NC, USA (E M McClure PhD, J L Moore MS); Department of Community Health, Aga Khan University, Karachi, Pakistan (S Saleem MBBS); Jawaharlal Nehru Medical College, Karnataka Lingayat Education University, Belagavi, India (S S Goudar MD); Department of Child Health and Paediatrics, Moi University School of Medicine, Eldoret, Kenya (F Esamai MBBS); Lata Medical Research Foundation, Nagpur, India (A Patel MD); Department of Pediatrics, University Teaching Hospital, University of Zambia, Lusaka, Zambia (E Chomba MD); Instituto de Nutrición de Centro América y Panamá (INCAP), Guatemala City, Guatemala (A L Garces MD); Department of Obstetrics and Gynecology, Columbia University, New York City, NY, USA (M S Harrison MD, R L Goldenberg MD); Department of Pediatrics, University of Colorado Anschutz Medical Campus, Denver, CO, USA (N F Krebs MD); Thomas Jefferson University, Philadelphia, PA, USA (R J Derman MD); Department of Pediatrics, University of Alabama at Birmingham, Birmingham, AB, USA (W A Carlo MD); Department of Pediatrics, Indiana University, Indianapolis, IN, USA (E A Liechty MD); Department of Global Health, Boston University School of Public Health, Boston University, Boston, MA, USA (P L Hibberd MD); and Tulane University School of Public Health and Tropical Medicine, New Orleans, LA, USA (P M Buekens MD)

## Abstract

**Background:**

Caesarean section prevalence is increasing in Asia and Latin America while remaining low in most African regions. Caesarean section delivery is effective for saving maternal and infant lives when they are provided for medically-indicated reasons. On the basis of ecological studies, caesarean delivery prevalence between 9% and 19% has been associated with better maternal and perinatal outcomes, such as reduced maternal land fetal mortality. However, the specific prevalence of obstetric and medical complications that require caesarean section have not been established, especially in low-income and middle-income countries (LMICs). We sought to provide information to inform the approach to the provision of caesarean section in low-resource settings.

**Methods:**

We did a literature review to establish the prevalence of obstetric and medical conditions for six potentially life-saving indications for which caesarean section could reduce mortality in LMICs. We then analysed a large, prospective population-based dataset from six LMICs (Argentina, Guatemala, Kenya, India, Pakistan, and Zambia) to determine the prevalence of caesarean section by indication for each site. We considered that an acceptable number of events would be between the 25th and 75th percentile of those found in the literature.

**Findings:**

Between Jan 1, 2010, and Dec 31, 2013, we enrolled a total of 271 855 deliveries in six LMICs (seven research sites). Caesarean section prevalence ranged from 35% (3467 of 9813 deliveries in Argentina) to 1% (303 of 16 764 deliveries in Zambia). Argentina’s and Guatemala’s sites all met the minimum 25th percentile for five of six indications, whereas sites in Zambia and Kenya did not reach the minimum prevalence for caesarean section for any of the indications. Across all sites, a minimum overall caesarean section of 9% was needed to meet the prevalence of the six indications in the population studied.

**Interpretation:**

In the site with high caesarean section prevalence, more than half of the procedures were not done for life-saving conditions, whereas the sites with low proportions of caesarean section (below 9%) had an insufficient number of caesarean procedures to cover those life-threatening causes. Attempts to establish a minimum caesarean prevalence should go together with focusing on the life-threatening causes for the mother and child. Simple methods should be developed to allow timely detection of life-threatening conditions, to explore actions that can remedy those conditions, and the timely transfer of women with those conditions to health centres that could provide adequate care for those conditions.

## Introduction

Caesarean sections are effective in saving maternal and infant lives when they are provided for medically-indicated reasons. However, there has been much debate about the appropriate population-based caesarean section prevalence. WHO has concluded that increases in caesarean sections of up to 10–15% of all births are associated with decreases in maternal, neonatal, and infant mortality.^1^ This assumption is based on ecological studies, which have shown that prevalences of 9–16% are associated with decreases in maternal, neonatal, and infant mortality.^2–5^ An ecological study involving 194 WHO member countries, published after the WHO recommendations, found that national caesarean section prevalence of up to about 19% of all deliveries were associated with lower maternal or neonatal mortality.^6^ Controversies arise when adjusting these associations by socioeconomic factors, suggesting that at caesarean section prevalence below 9–16% of all births, socioeconomic development might be the major determinant for mortality rather than the prevalence of caesarean sections.^5^

Studies have shown that 24% of countries in the world which account for nearly a quarter of the total number of births worldwide (29·5 million) have fewer than 5% by caesarean section.^7^ One estimation of caesarean section trends^8^ showed that in the past 24 years, the prevalence of caesarean section has had minimal change (from 2·3% to 3·5%) in sub-Saharan Africa.

The objective of our analysis is to inform the initiatives aimed at addressing the availability and consequences of caesarean section in low-resource settings. We believe that the use of caesarean section should address life-threatening events and the overall approach should be to do the fewest caesarean sections that would be sufficient to address life-threatening events. To contribute to this approach, we reviewed the literature including the frequency of life-threatening events and the prevalence of caesarean section due to these events in low-resource settings. Next, we analysed a multi-country research network dataset of communities in six low-income and middle-income countries (LMICs) to assess the use of caesarean section and their indications. The study was completed as part of the Global Network for Women’s and Children’s Health Research (Global Network), a multi-country research network in LMICs.^9^

## Methods

### Hypothesis

We framed our study analyses based on the assumption that first, there are several conditions that require a caesarean section delivery to save the maternal, fetal, or infant life. These conditions, referred to in this paper as life-saving indications, are cord prolapse or fetal distress; major antepartum haemorrhage; obstructed labour; severe pre-eclampsia or eclampsia; transverse, oblique lie, or breech presentation; and uterine rupture. Second, the distribution of each life-saving indication’s prevalence can be established from the literature and compared to a country’s expected caesarean section prevalence by indication. We considered that an acceptable caesarean section prevalence would be between the 25th and 75th percentile on the basis of the literature review. Although the selection of these cutoff points was an arbitrary decision, we took this approach on the basis of the notion that prevalence below the 25th percentile were considered too low and prevalence above the 75th percentile were considered too high to achieve optimal outcomes.

### Literature review

We did a literature review on the frequency of life-threatening events and the prevalence of caesarean section for life-saving indications in clinical studies.^10–29^ We first searched the PubMed literature since 1980 for the terms “cesarean section” and “indications” including cord prolapse, fetal distress, major antepartum haemorrhage, obstructed labour, pre-eclampsia, malpresentation, and uterine rupture. We focused the assessment on the six major conditions that are included in the prospective data collection in the Global Network of Maternal and Neonatal Health (cord prolapse or fetal distress; major antepartum haemorrhage; obstructed labour; severe preeclampsia or eclampsia; transverse, oblique lie, or breech presentation; and uterine rupture).

### Global Network Maternal and Newborn Health Registry (MNHR)

Next, we used descriptive analyses from the Global Network’s MNHR. The Global Network MNHR is a multi-country research study designed to obtain outcomes for all pregnancies within defined geographic regions (clusters) in six LMICs (Argentina, Guatemala, India [two sites], Pakistan, Kenya, and Zambia).^30^ These communities are in low-resource settings in semi-urban and rural areas. Trained registry administrators gathered data on pregnancies, deliveries, and neonates from pregnant women residing within the clusters in six LMIC countries. The sites, a total of 101 clusters, were geographically-defined catchment areas serving one to three health centres and delivering an average of 300 to 500 annual births. The study population included all births that occurred within the catchment site, regardless of delivery location.

For the MNHR, all women were registered during pregnancy by study staff and, following written consent, a follow-up visit was done after delivery and at 6-weeks postpartum. At the delivery follow-up visit, a brief survey was completed which defined maternal, fetal, and neonatal complications during pregnancy, delivery, and until 42 days postpartum. The major complications obtained for all women included obstructed or prolonged labour; preeclampsia or eclampsia; transverse or oblique lie, and antepartum haemorrhage. For women who were delivered by caesarean section, the clinician-defined indication for the procedure was also recorded and categorised on the basis of predefined indications for caesarean section published by Stanton and colleagues.^31^ The MNHR study was approved by the institutional review boards and ethics review committees at all participating institutions.

### Statistical analysis

The specific proportion of caesarean section procedures per life-saving indication were extracted directly from the literature, if provided, or calculated with the available data by use of the following formula:

$$\text{Specific ceasarean prevalence }=\frac{\text{ Number of ceasarean sections per life-saving indication }}{\text{ Total number of deliveries }}$$

For example, Kolas and colleagues^19^ investigated the indications for caesarean section in Norway in 24 157 deliveries and found that 178 were due to preeclampsia or eclampsia. Using the formula given above, the specific caesarean section prevalence for that life-saving indication was 178 indications (1%) of 24 157 deliveries. That same formula was applied for each life-saving indication present in the selected studies. Using these figures, we estimated the 10th, 25th, 50th, and 75th percentiles to define the frequency of these life-saving indications and the prevalence of caesarean section done under these indications. We then assumed that for determining the safe number of caesarean sections, the caesarean section prevalence by indication should be at or above the 25th percentile for each life-saving indication and ideally not exceed the 75th percentile values. We analysed the caesarean section prevalence from the Global Network’s MNHR database by life-saving indication using all women enrolled who received a caesarean section and the indication for each procedure. Data analyses included descriptive statistics and were performed in SAS version 9.3.

### Role of the funding source

The funder of the study had no role in study design, data collection, data analysis, data interpretation, or writing of the report. The corresponding author had full access to all the data in the study and had final responsibility for the decision to submit for publication.

## Results

Because few studies (n=15) were identified in the initial PubMed search, the search was expanded to include the grey literature, primarily reports from hospitals, health agencies, and ministries of health using Google Scholar for which an additional five studies met the criteria. Altogether, 20 studies were identified and included in the analyses (table 1).

From the selected studies, eight included data from hospital or national databases. We also included three prospective studies, three cross-sectional studies, one retrospective cohort, and one academic report. The remaining four reports were national surveys done by governmental or non-governmental health organisations. Of the 20 studies identified and included, seven were from Asia (Indonesia, Philippines, Malaysia, Thailand, Pakistan, India, and Nepal), four from Europe (Norway, Sweden, Portugal, and the UK), two represented different countries across the African continent (Democratic Republic of Congo, Burundi, and Sierra Leone), one study was performed in Australia, and six were from the Americas (five from North America [USA and Canada] and one in Central America [El Salvador]).^10–29^

A total of 271 855 deliveries were registered by the Global Network’s MNHR between 2010 and 2013. Baseline characteristics show that women without formal schooling ranged from 254 (3%) of 9901 women in sites in Argentina, 1204 (3%) of 39 250 women in Nagpur (India), and 1089 (3%) of 35 621 women in Kenya, to 41 007 (83%) of 49 550 women in Pakistan. Home births ranged between 83 (1%) of 9901 births in Argentina and 20 769 (58%) of 35 621 births in Kenya, whereas caesarean sections occurred in 550 (1%) of 14 851 births in Kenya and in 1331 (1%) of 16 764 births in Zambia and in 3467 (35%) of 9813 births in the Argentinian sites (table 2). In Kenya, 14 851 (42%) of 35 621 deliveries were either in a clinic or a hospital, which—together with Guatemala, for which 13 414 (44%) of 30 259 deliveries were in health facilities—represented the lowest prevalence of deliveries in health facilities.

The frequency of complications that we considered life-saving indications for a caesarean section was similar between most sites, except for the Pakistan site, which had a much higher frequency of reported complications compared with the other sites (table 3). For example, the prevalence of major antepartum haemorrhage ranged from 1% to 5%, obstructed or prolonged labour varied between 4% and 11%, hypertensive disorders (including severe pre-eclampsia and eclampsia) between 1% and 7% and fetal malpresentation (breech, transverse, or oblique lie) between 1% and 4%.

In the Argentinian site, where the highest occurrence of caesarean section was observed, having a previous caesarean section accounted for 36% of the indications (table 4), even though it is not considered as a life-threatening cause by this study on the basis of the literature.^31^ A minor proportion of caesarean sections were done because of a previous caesarean section in the sites with lower caesarean section occurrence. Moreover, caesarean section by maternal request was an indication for 6% of the caesarean sections at the Argentinian site and almost never an indication at the other sites.

The frequency of indications that were considered as life-saving indications for performing a caesarean section and the calculated 10th, 25th, 50th and 75th percentiles from the literature review for each indication are shown in table 5. The countries that failed to achieve the 25th percentile of caesarean section by indication, reported by site are highlighted. As shown, the site with the highest caesarean section prevalence, the Argentinian site (35%), accomplished the same amount of caesarean section for life-saving indications over the 25th percentile (five of six) as the Guatemalan site with a caesarean section prevalence of about half of the prevalence at the Argentinian site (18%). The Nagpur (India; caesarean section occurrence of 20%), Belgaum (India; 14%), and Pakistan sites (9%) had only three of the six life-saving indications above the 25th percentile, while the Kenyan (2%) and Zambian (1%) sites did not reach the 25th percentile for any indication.

The 75th percentile of indications for caesarean section showed variable results. For example, the Argentinian site, with the highest overall caesarean section prevalence, exceeded the 75th percentile for three of the six indications. In the Indian sites, the diagnosis of obstructed or prolonged labor was well above the 75th percentile of the expected values, whereas in the Guatemalan site, one of the six indications was above the 75th percentile of the expected values.

Focusing on the percentage of caesarean sections that were performed for conditions other than those selected as life-saving indications, in the Guatemalan site the caesarean section prevalence for other indications was lower than in the Argentinian site. This suggests an excess of unnecessary caesarean section procedures when the global caesarean section prevalence surpasses 18%. Data from the Guatemalan site show that a caesarean section prevalence of 9·6% is attributed to life-saving indications whereas 8·9% is due to other indications, predominately previous caesarean section (table 4).

## Discussion

This study provides information that might help in setting a safe minimum proportion of caesarean sections in LMICs that would cover the frequency of selected life-saving indications. Similar coverage of these indications was achieved in Argentina with 99% of hospital deliveries and an overall caesarean section prevalence of 35% by contrast with Guatemala, which had an overall prevalence of caesarean section of 18% and 44% of hospital deliveries. The overall prevalence of caesarean section was low for the African sites and did not reach the minimum necessary number of caesarean sections for any of the life-saving indications. This was an expected result since the sum of the expected life-saving indications was at least 4%. Across the sites, we observed a wide range of the frequency of life-saving indications for caesarean sections, with some sites having a very low prevalence of indications.

This analysis presents population-based data on deliveries from a range of culturally-diverse countries with varying sociodemographic characteristics, in an attempt to define a safe proportion of caesarean sections to address the concerning inequity in the distribution of caesarean sections worldwide. One of the major strengths of this study is that it provides population-based information from settings in LMICs including numerous communities at each study site, many of which had a high proportion of home deliveries with few participants lost to follow-up. Weaknesses of the study include that there was no validation of the cause of caesarean section because the data were based on the health-care provider’s reports. Therefore, there might be bias in these data, especially from African sites because of under-reporting of conditions, given the relatively high numbers of home deliveries. The causes of caesarean section, namely cord prolapse and fetal distress, were categorised together in the MNHR, making it impossible to discriminate the frequency of caesarean section for each factor separately. The diagnosis of fetal distress might vary by provider or the technology used, such as the availability of electronic fetal monitoring. Other relevant limitations include the assumptions regarding the life-threatening causes of caesarean sections that were considered life-saving. It can be argued that breech presentation is not a life-saving indication but, unfortunately, we were unable to disentangle breech from other malpresentations because they were also included as a joint category during data collection.

Defining appropriate indications for caesarean section has been one approach to identifying the proportion of caesarean sections necessary to save a maternal, fetal, and neonatal life.^30^ Another approach has been the Robson classification system,^32^ which does not address indication, but instead uses obstetric parameters such as pregnancy history and gestational age to divide women having caesarean sections into ten categories. This system has primarily been used to compare caesarean section trends over time and across settings rather than to define the minimum procedures needed to save lives. Use of the Robson criteria has potential limitations in LMICs, where obstetric parameters including gestational age might not be reliable.^32,33^ Future research related to the Robson classification might include obtaining a better understanding of the indications for caesarean sections within each of the ten categories.

Overall, further research should include a strong attempt to identify accurate data on the indications for caesarean sections, focusing on those that are life-threatening. Standardisation of the definitions for life-threatening conditions will be important for comparison of studies over time and in different locations. We also understand that the conditions that are included as life-threatening are somewhat arbitrary. For example, although breech presentations are often considered life-threatening for the fetus, in actuality, the risk is relatively low and in some areas might not be considered a life-threatening condition. As long as the conditions considered life-threatening are specified, it is reasonable to use those conditions to define a minimum acceptable proportion of caesarean sections for a given population. We also understand that in assessing the appropriate use of caesarean section, the condition for which it is performed is not the only criterion to be used. Time-liness of the surgery, for example, would be another consideration.

This approach for the identification of the major life-threating conditions might inform the various actions that, together with appropriate caesarean section, might reduce mortality in LMICs. As an example, one approach that might be feasible in low-resource settings is use of simple, portable ultrasound equipment that can be used in community settings for early screening for life-threatening events and early referral. In this example, detection of a transverse, oblique lie, or breech presentation could be followed by a caesarean section thus avoiding the consequences of an obstructed labour.^34^ As another example, implementation research is needed for the potential for cell phones to assist with early diagnosis and identification of women with a life-threatening event requiring a caesarean section.

Regarding the approach to define an expected safe minimum proportion of caesarean section, we believe that it should be based on indications that imply a threat to the mother’s or the fetus’s life. No association analysis between caesarean section and maternal and perinatal mortality was done because of the low frequency of these events in the dataset used. However, this approach suggested a range of different scenarios worth exploring. On the one hand, caesarean sections with a prevalence as low as 2% were insufficient to provide the procedure for any of the life-saving indications; on the other hand, in several sites with high caesarean section prevalence, a high proportion of caesarean sections were not done for one of the life-saving indications. For example, in Argentina, with a 35% prevalence of caesarean section, only 44% of the procedures were done in response to a life-saving indication, whereas 56% were done for not completely justified reasons. In another scenario, Guatemala had only 44% of deliveries in a medical facility and had a prevalence of caesarean section of 18% for which only 53% of these procedures were done for one of the life-saving indications. This finding suggests that although the overall prevalence of caesarean section was acceptable, there was a proportion of the population who delivered outside the health-care system and therefore did not have access to life-saving caesarean section. Many of the sites had estimates of events, such as prolonged labour, that were well above the expected prevalence. There is great concern about the over-diagnosis of various obstetric conditions and the potential for adverse consequences associated with poorly justified caesarean section, particularly in low-resource settings. Women who receive an unnecessary caesarean section also have increased risks for future pregnancies. Research focused on methods to improve accuracy of diagnosis of prolonged labour is needed to avoid unnecessary procedures and their associated risks.

Results of these analyses showed some similarities with previous ecological studies.^2–6^ With prevalence of caesarean sections as high as 35%, more than half of the completed procedures were not caused by complications during childbirth. Prevalences of 18% are closer to covering the frequency of life-saving conditions, also reducing the frequency of caesarean section deliveries for causes not justified. Values below 9% did not achieve the expected frequency of life-saving conditions. Accounting for the proportion of home deliveries, the Guatemalan site, which had 56% of deliveries done at home, could achieve 18% of caesarean section, covering five of six life-saving conditions, whereas the African sites, which had a similar proportion of home deliveries, only reached 1–2% of caesarean section births, and did not cover the expected prevalences of life-saving conditions of caesarean sections.

In conclusion, this article is an attempt to contribute to the discussion of identifying appropriate caesarean section prevalence in low-resource settings. Our suggestion is that attempts to establish an appropriate minimum number of procedures per population should go hand in hand with focusing on the life-threatening causes for the mother and child. Simple methods should be developed to allow timely detection of life-threatening conditions, to explore actions that can remedy those conditions, and the timely transfer of women with those conditions to health centres that could provide adequate care for those conditions.

## Figures and Tables

**Table 1:** Proportion of caesarean sections for life-saving indications in clinical studies

	Cord prolapse or fetal distress	Major antepartum haemorrhage	Obstructed labour	Severe pre-eclampsia or eclampsia	Transverse, oblique lie, or breech presentation	Uterine rupture	Total life-threatening conditions
Number of sites included	13 sites	10 sites	21 sites	15 sites	22 sites	7 sites	..
Percentile							
10^th^ percentile	0·1%	0·2%	0·7%	0·3%	0·3%	0·1%	1·7%
25^th^ percentile	0·3%	0·4%	1·9%	0·5%	1·0%	0·2%	4·3%
50^th^ percentile	1·2%	0·6%	2·5%	0·8%	2·2%	0·4%	7·6%
75^th^ percentile	3·3%	1·6%	4·6%	1·5%	4·4%	2·4%	17·7%

On the basis of literature review.^9,28^

**Table 2:** Characteristics of women in the Maternal Newborn Health Registry, Global Network sites 2010–13

	Latin America		Asia			Africa	
	Argentina	Guatemala	Nagpur, India	Belgaum, India	Pakistan	Kenya	Zambia
Total deliveries	9901	30 259	39 250	79 674	49 550	35 621	27 600
Maternal age							
<20 years	2678 (27%)	4939 (16%)	771 (2%)	7621 (10%)	1898 (4%)	7767 (22%)	6975 (25%)
20–35 years	6428 (65%)	22 090 (73%)	38 342 (98%)	71 838 (90%)	44 728 (91%)	26 278 (74%)	18 397 (67%)
36+ years	758 (8%)	3214 (11%)	108 (<1%)	137 (<1%)	2752 (6%)	1507 (4%)	2178 (8%)
Maternal education							
No formal schooling	254 (3%)	5882 (19%)	1204 (3%)	16 399 (21%)	41 007 (83%)	1089 (3%)	2866 (11%)
Primary	6131 (63%)	19 104 (63%)	6814 (17%)	26 254 (33%)	3749 (8%)	25 368 (71%)	15 091 (55%)
Secondary	3246 (33%)	4928 (16%)	23 335 (60%)	29 101 (37%)	2910 (6%)	7817 (22%)	8966 (33%)
University	152 (2%)	330 (1%)	7848 (20%)	7306 (9%)	1681 (3%)	1287 (4%)	492 (2%)
Parity							
0	3224 (33%)	8425 (28%)	18 898 (48%)	33 823 (43%)	10 224 (21%)	8937 (25%)	7453 (27%)
1	2373 (24%)	6354 (21%)	15 873 (41%)	27 534 (35%)	8489 (17%)	7646 (22%)	5615 (20%)
2 or more	4232 (43%)	15 475 (51%)	4470 (11%)	17 819 (23%%)	30 706 (62%)	18 979 (53%)	14 495 (53%)
At least one antenatal care visit							
Yes	9311 (95%)	29 694 (98%)	39 204 (100%)	79 401 (100%)	41 679 (85%)	34 596 (97%)	27 420 (99%)
No	494 (5%)	533 (2%)	18 (<1%)	67 (<1%)	7638 (16%)	1001 (3%)	158 (1%)
Birth location							
Hospital	9788 (99%)	12 047 (40%)	26 635 (68%)	53 878(68%)	14 362 (29%)	4613 (13%)	3435 (12%)
Clinic	25 (<1%)	1367 (5%)	10 809 (28%)	20 636 (26%)	12 305 (25%)	10 238 (29%)	13 329 (48%)
Home/Other	83 (1%)	16 844(56%)	1781 (5%)	5097 (6%)	22 821 (46%)	20 769 (58%)	10 833 (39%)
Birth attendant							
Physician	7162 (72%)	12 949 (43%)	23 470 (60%)	46 814 (59%)	12 710 (26%)	708 (2%)	631 (2%)
Nurse or midwife	2678 (27%)	543 (2%)	14 178 (36%)	28 275 (36%)	13 136 (25%)	14 488 (41%)	15 328 (56%)
Traditional birth attendant	2 (<1%)	16 674 (55%)	1108 (3%)	1950 (3%)	22 312 (45%)	16 022 (45%)	7070 (26%)
Family or unattended	51 (1%)	92 (<1%)	471 (1%)	2611 (3%)	1343 (3%)	4401 (12%)	4568 (17%)
Caesarean section							
Yes	3467 (35%)	5576 (18%)	7697 (20%)	11 218 (14%)	4632 (9%)	550 (2%)	303 (1%)
No	6346 (65%)	24 683 (82%)	31 553 (80%)	68 456 (86%)	44 918 (91%)	35 071 (98%)	27 297 (99%)

Parity was defined as pregnancies at least 20 weeks previously, excluding the current pregnancy.

**Table 3:** Total deliveries, facility deliveries, and reported obstetric complications by Global Network site, 2010–13

	Total deliveries	Facility deliveries	Major antepartum haemorrhage	Obstructed or prolonged labour	Severe pre-eclampsia or eclampsia	Transverse, oblique lie, or breech presentation
Argentina	9901	9813 (99%)	68 (1%)	793 (8%)	378 (4%)	205 (2%)
Guatemala	30 259	13 414 (44%)	504 (2%)	2151 (7%)	1212 (4%)	939 (3%)
Nagpur, India	39 250	37 444 (95%)	225 (1%)	4177 (11%)	854 (2%)	1100 (3%)
Belgaum, India	79 674	74 514 (94%)	531 (1%)	8218 (10%)	1731 (3%)	1107 (1%)
Pakistan	49 550	26 667 (54%)	2393 (5%)	9943 (20%)	3307 (7%)	1795 (4%)
Kenya	35 621	14 851 (42%)	837 (2%)	3820 (11%)	579 (2%)	575 (2%)
Zambia	27 600	16 764 (61%)	339 (1%)	1174 (4%)	242 (1%)	281 (1%)

Data are n or n (%).

**Table 4:** Caesarean sections and specific indications for caesarean section by Global Network site, 2010–13

	Deliveries	Caesarian section	Cord prolapse or fetal distress	Major antepartum haemorrhage	Obstructed or prolonged labour	Severe pre-eclampsia or eclampsia	Transverse, oblique lie, or breech presentation	Uterine rupture	Previous caesarian section	Previous fistula repair	Maternal request	Other	No clear indication	No data
Argentina	9901	3467 (35%)	60 (1%)	68 (1%)	793 (8%)	378 (4%)	205 (2%)	19 (<1%)	1245 (13%)	3 (<11%)	204 (2%)	173 (2%)	127 (1%)	192 (6%)
Guatemala	30 259	5576 (18%)	364 (1%)	57 (<1%)	1234 (4%)	382 (1%)	788 (3%)	75 (<1%)	1337 (4%)	30 (<1%)	66 (<1%)	844 (3%)	300 (1%)	99 (2%)
Nagpur,lndia	39 250	7697 (20%)	504 (1%)	64 (<1%)	3608 (9%)	268 (1%)	953 (2%)	17 (<1%)	1209 (3%)	16 (<1%)	67 (<1%)	338 (1%)	649 (2%)	4 (<1%)
Belgaum, India	79 674	11 218 (14%)	307 (<1%)	175 (<1%)	5831 (7%)	363 (1%)	893 (1%)	67 (<1%)	1782 (2%)	14 (<1%)	230 (<1%)	495 (1%)	195 (<1%)	866 (8%)
Pakistan	49 550	4632 (9%)	222 (1%)	183 (<1%)	2291 (5%)	120 (<1%)	532 (1%)	18 (<1%)	1017 (2%)	24 (<1%)	76 (<1%)	44 (<1%)	20 (<1%)	88 (2%)
Kenya	35 621	550 (2%)	21 (<1%)	19 (<1%)	321 (1%)	17 (<1%)	93 (<1%)	3 (<1%)	59 (<1%)	0	1 (<1%)	11 (<1%)	2 (<1%)	3 (<1%)
Zambia	27 600	303 (1%)	8 (<1%)	67 (<1%)	2 (1%)	9 (<1%)	36 (<1%)	3 (<1%)	26 (<1%)	0	1 (<1%)	3 (<1%)	14 (<1%)	33 (11%)

Data are n or n(%) unless otherwise indicated. The specific prevalence of caesarean sections per life-saving indication was calculated dividing the number of caesarean sections per life-saving indication per site over the total number of deliveries per site

**Table 5:** Percentage of life-saving indications for caesarean section and number of indications above the 10th, 25th, 50th, and 75th percentiles

	Caesarian section rate	Cord prolapse or fetal distress	Major antepartum haemorrhage	Obstructed or prolonged labour	Severe pre-eclampsia or eclampsia	Transverse, oblique lie, or breech presentation	Uterine rupture	Total number of caesarean sections for life-saving indications	Total number of caesarean sections for other indications	Number of indications abovethe 10th percentile	Number of indications abovethe 25th percentile	Number of indications abovethe 50th percentile	Number of indications above the 75th percentile
Argentina	3467 (35%)	60 (1%)	61 (1%)	792 (8%)*	316 (3%)*	295 (3%)	19 (<1%)†	1543 (15%)	1924 (20%)	6	5	3	3
Guatemala	5576 (18%)	364 (1%)	57 (<1%)†	1234 (4%)	382 (1%)	788 (3%)	75 (<1%)	2900 (10%)	2676 (9%)	5	5	4	1
Nagpur, India	7697 (20%)	504 (1%)	64 (<1%)†	3608 (9%)*	268 (1%)	953 (2%)	17 (<1%)†	5414 (14%)	2283 (6%)	4	4	3	2
Belgaum, India	11 218 (14%)	307 (<1%)	175 (<1%)†	5831 (7%)	363 (<1%)†	893 (1%)	67 (<1%)†	7636 (10%)	3582 (4%)	5	3	1	2
Pakistan	4632 (9%)	222 (<1%)	183 (<1%)†	2291 (5%)*	120 (<1%)†	532 (1%)†	18 (<1%)†	3366 (7%)	1266 (3%)	4	3	1	2
Kenya	550 (2%)	21 (<1%)†	19 (<1%)†	321 (1%)†	17 (<1%)†	93 (<1%)†	3 (<1%)†	474 (1%)	76 (<1%)	1	0	0	0
Zambia	303 (1%)	8 (<1%)†	67 (<1%)†	2 (<1%)†	9 (<1%)†	36 (<1%)†	3 (<1%)†	125 (1%)	178 (<1%)	1	0	0	0
25th-75th percentiles	4·34–17·66%	0·25–3·30%	0·42–1·58%	1·91–4·58%	0·53–1·45%	1·03–4·35%	0·20–2·40%	..	..	..	..	..	..

Data are n or n (%) unless otherwise indicated. This table provides information on the number of indications above the 25th percentile accomplished by each site. Argentina and Guatemalan sites accomplished five of six indications above the 25th percentile, whereas Kenya and Zambia, with a low overall caesarean section prevalence did not accomplish the 25th percentile for any indications.

* Indications above the 75th percentile.

† Indications below the 25th percentile

## Research in context

### Evidence before this study

We did a literature review on the frequency of life-threatening events and the rates of caesarean section for life-saving indications in clinical studies. We first searched the PubMed literature since 1980 for the terms “cesarean section” and “indications” including cord prolapse, fetal distress, major antepartum haemorrhage, obstructed labour, pre-eclampsia, malpresentation, and uterine rupture. On the basis of ecological studies, a caesarean section prevalence between 9·19% was associated with lower maternal and neonatal mortality compared with countries with a prevalence of less than 5%. Controversies arise when adjusting these associations by socioeconomic factors, suggesting that at caesarean section prevalence below 9%, socioeconomic development might be the major determinants for mortality rather than the proportion of procedures completed.

### Added value of this study

Our study assessed the appropriate lower caesarean section prevalence based on the expected proportions per indication of life-saving conditions in low-resource settings. The analysis of the proportion of caesarean sections for life-saving conditions in low-income and middle-income settings could inform the actions needed to focus on those conditions for which caesarean section are not currently provided at the accepted values.

### Implications of all the available evidence

Attempts to establish a minimum prevalence of caesarean section should go together with focusing on the life-threatening causes for the mother and child. Simple methods should be developed to allow timely detection of life-threatening conditions, to explore actions that can remedy those conditions, and the timely transfer of women with those conditions to health centres that could provide adequate care for those conditions.
